# Trehalose Improves Human Fibroblast Deficits in a New CHIP-Mutation Related Ataxia

**DOI:** 10.1371/journal.pone.0106931

**Published:** 2014-09-26

**Authors:** Maria Jose Casarejos, Juan Perucho, Jose Luis López-Sendón, Justo García de Yébenes, Conceição Bettencourt, Ana Gómez, Carolina Ruiz, Peter Heutink, Patrizia Rizzu, Maria Angeles Mena

**Affiliations:** 1 Department of Neurobiology, Hospital Ramón y Cajal, Madrid, Spain; 2 Department of Neurology, Hospital Ramón y Cajal, Madrid, Spain; 3 CIBERNED, Instituto de Salud Carlos III, Madrid, Spain; 4 Department of Molecular Neuroscience, UCL Institute of Neurology, London, United Kingdom; 5 Institute for Molecular and Cell Biology (IBMC), University of Porto, Porto, Portugal; 6 Center of Research in Natural Resources (CIRN) and Department of Biology, University of the Azores, Ponta Delgada, Portugal; 7 German Center for Neurodegenerative Diseases, Tübingen, Germany; University of Florida, United States of America

## Abstract

In this work we investigate the role of CHIP in a new CHIP-mutation related ataxia and the therapeutic potential of trehalose. The patient's fibroblasts with a new form of hereditary ataxia, related to *STUB1* gene (CHIP) mutations, and three age and sex-matched controls were treated with epoxomicin and trehalose. The effects on cell death, protein misfolding and proteostasis were evaluated. Recent studies have revealed that mutations in STUB-1 gene lead to a growing list of molecular defects as deregulation of protein quality, inhibition of proteasome, cell death, decreased autophagy and alteration in CHIP and HSP70 levels. In this CHIP-mutant patient fibroblasts the inhibition of proteasome with epoxomicin induced severe pathophysiological age-associated changes, cell death and protein ubiquitination. Additionally, treatment with epoxomicin produced a dose-dependent increase in the number of cleaved caspase-3 positive cells. However, co-treatment with trehalose, a disaccharide of glucose present in a wide variety of organisms and known as a autophagy enhancer, reduced these pathological events. Trehalose application also increased CHIP and HSP70 expression and GSH free radical levels. Furthermore, trehalose augmented macro and chaperone mediated autophagy (CMA), rising the levels of LC3, LAMP2, CD63 and increasing the expression of Beclin-1 and Atg5-Atg12. Trehalose treatment in addition increased the percentage of immunoreactive cells to HSC70 and LAMP2 and reduced the autophagic substrate, p62. Although this is an individual case based on only one patient and the statistical comparisons are not valid between controls and patient, the low variability among controls and the obvious differences with this patient allow us to conclude that trehalose, through its autophagy activation capacity, anti-aggregation properties, anti-oxidative effects and lack of toxicity, could be very promising for the treatment of CHIP-mutation related ataxia, and possibly a wide spectrum of neurodegenerative disorders related to protein disconformation.

## Introduction

Hereditary ataxias constitute a group of rare neurological disorders (prevalence from 5 to 6, 5 per 100,000) [1], which are clinically and genetically heterogeneous. Core neurological deficits are characterized by a progressive cerebellar syndrome with imbalance, unsteady gait and a lack of limb coordination, dysarthria and eye movement abnormalities. The majority are autosomal dominant (AD) and autosomal recessive (AR). For AD ataxias, known as spinocerebellar ataxias (SCAs), more than 20 causative genes have been identified. The AR ataxias are even more diverse involving nearly 100 genes.

According to a recent study [2], we and others have identified variants in the *STUB1* gene, which encodes for the C-terminal HSP70-interacting protein (CHIP), in patients with ataxia of unknown cause [3] (Bettencourt et al, currently submitted). We investigated the molecular changes occurring in fibroblasts of a patient from the Spanish ataxia family, who has a pattern of transmission consistent with autosomal recessive inheritance. The two affected members of the family were compound heterozygous for two mutations in the *STUB1* gene which translate into one stop codon, p.Glu238Ter, and one amino acid change, p.Met211Ile, in the protein sequence. CHIP is a co-chaperone and ubiquitin ligase protein involved in protein-processing, and those two changes are located in protein regions critical for its function. The first one is in the U-Box domain, critical for dimerization of the protein and binding to ubiquitin, and the second mutation is in one of the residues of the second dimer interface [4], [5]. Several studies have proved that CHIP mutations in mice and human fibroblast lead to reduced lifespan and accelerated age-related phenotypes, like cellular senescence and increased indices of oxidative stress [6]–[8].

Trehalose is a natural disaccharide that protects cells from various stressing environmental factors. These effects could be explained by its protective properties as an inducer of autophagy and chemical chaperone and may be relevant for the treatment of different neurodegenerative diseases [9]. Trehalose inhibits amyloid formation *in vitro* and prevents aggregation of beta-amyloid in models of Alzheimer's disease (AD) [10], [11]. It has recently been shown that trehalose inhibits polyglutamine-mediated aggregation in *in vitro* and *in vivo* models of Huntington's disease (HD) [12], [13]. Furthermore, trehalose accelerates the clearance of mutant huntingtin and α-synuclein and inhibits protein misfolding [12], [14]. Epoxomicin is a cell-permeable, natural product that selectively and irreversibly inhibits proteasome activity. Epoxomicin modifies four catalytic sub-units of the 20S proteasome, resulting primarily in inhibition of the chymotrypsin-like activity [15].

We have already shown that, in different models of abnormal proteasome function, the enhancement of autophagy by treatment with trehalose compensates for the biochemical and pathological deficits [16]–[18]. In this study, we investigated the deficits in baseline, stressing conditions through proteasome inhibition by epoxomicin and the effects of trehalose in the fibroblasts of an ataxia patient with mutant CHIP.

## Methods

### Ethics Statement

Written informed consent was obtained from the next of the kin of the subject, and according to the Spanish Law of Biomedical Research 14/2007 this is sufficient to perform the investigation.

This study was previously approved by the Ethical Committee: IRB00009134 (Hospital Universitario Ramón y Cajal de Madrid IRB #1) and IRYCIS (FWA00019802) IRB Federalwide Assurance (FWA) for the Protection of Human Subjects, registry number: IRB00009134 (CEIC).

### Drugs, culture media, chemical reagents

Epoxomicin, Suc-Leu-Leu-Val-Tyr-AMC and trehalose dyhidrate were purchased from Calbiochem (Darmstadt, Germany). The 20S Proteasome activity assay kit was from Chemicon (Temecula, CA, USA). Culture media and antibodies were used as previously described [19], [20]. 5,5-Dithio-bis-2-nitrobenzoic acid (DTNB), reduced (GSH) form of glutathione and chloroquine (CQ) were from Sigma (Madrid, Spain). The BCA protein assay kit was from Pierce (Rockford, Ill, USA). All other reagents were the purest that were commercially available from Merck or Sigma.

### Isolation and culture of primary fibroblasts

After obtaining informed consent from the next of the kin, a skin biopsy from the radial portion of the proximal forearm was performed on one patient with CHIP deficient ataxia and in three age and sex-matched voluntary controls. Skin specimens were placed in a 10 cm Petri dish with PBS and were cut into approximately 1 cm^2^ pieces. Then, 4 or 5 explants were placed into 25 cm^2^ flask cultures for 15–20 minutes in a humidified incubator at 37°C and 5% CO_2_ in air to promote its attachment. After that, 10 ml of growth medium Amniomed Plus (Genycell Biotech, Spain), a complete medium with foetal bovine serum (FBS), antibiotics and other ingredients were added and incubated for 1–2 weeks. The medium was exchanged every 5 days and when sufficient cells had migrated from the explants, cultures were trypsinizated with 0.2% trypsin (Sigma-Aldrich, Spain) made up of F-12 medium without FBS for 5 minutes and transferred to a new flask. This process was repeated until the desired amount of fibroblasts was obtained or until no further outgrowth was observed.

All studies were performed with human fibroblasts at early passages (7–9 passages). For cell proliferation and GSH studies fibroblast from later passages (12 passages) were used. The cultures were seeded at a density of 10^4^ cells/cm^2^ in glass cover slides pre-coated with poly-D-lysine (4.5 ug/cm^2^) or in 12- well plates and grown in Dulbecco's modified Eagle's medium containing 10% fetal calf serum and 50 U/ml penicillin/streptomycin (Gibco, Life-Technologies, Paisley, Scotland, UK) for 3–4 days, before being treated in the confluent state.

### Cell viability measurements

Fibroblast survival was measured analyzing the percentage of cells immunoreactive to cleaved caspase-3 [21]. Cultures were fixed with 4% paraformaldehyde, washed in 0.1 M phosphate-buffered saline, pH 7.4 (PBS), permeabilized with ethanol-acetic acid (19∶1), and incubated at 4°C for 24 h with a rabbit polyclonal anti-cleaved caspase-3 (1/400) from Cell-Signaling Technology (Boston, MA, USA). Cells were then washed ×3 in PBS and incubated with anti-mouse Alexa Fluor 488 (Green) for 1 h at room temperature. After the final 3 washes with PBS, cover slips were mounted in anti-fading solution, 3×10^−6^ M final concentration and viewed under a flourescent microscope (Nikon C1 plus ECLIPSE Ti-e microscope). To assess antibody specificity of cleaved caspase-3, negative control was included by omission of the primary antibody.

To assay the cell proliferation index of cells, 48-hours after seeding cell cultures were incubated with 50 mM BrdU 24 h before fixation and permeabilization (ethanol/acetic acid solution and 2 N HCl). For immunodetection, we used a mouse anti-BrdU antibody (1/20) and anti-mouse Ig-fluorescein antibody. Nuclei were stained by bis-benzimide (Hoechst33342) and immunostaining was visualized under fluorescent microscopy. The quantification of immunoreactive cells was analyzed in predefined parallel strips. Additional explanations to the methods used are shown in every figure with immunohistochemistry data.

### Measurement of Reactive Oxygen Species (ROS) production and GSH

ROS production by quantification of DCF^+^ cells was previously described in [21]. The abundance of reactive oxygen species (ROS) was determined by using 2′,7′-dichlorofluorescein diacetate (DCFH-DA). Control and CHIP mutant fibroblasts were seeded in glass coverslips, pre-coated with poly-D-lysine, three days before the experiment. After 15 min of treatment with trehalose (100 mM), the cells were incubated with the proteasome inhibitor epoxomicin (5 nM) for another 24 hours. After this time, the cells were washed twice, and incubated with 5 µM DCFH-DA in EMEM free of phenol red for 30 min, at 37°C in the incubator. Then, cells were washed twice with (PBS with 1 mM glucose), and the nuclei were stained with bis-benzimide (Hoechst 33342) added to the anti-fading solution, at a 3×10^−6^ M final concentration. For quantitative determinations, the cover slides were observed under fluorescent microscope using FITC filter and 150 to 200 cells were counted in predefined parallel strips; ROS positive cells were identified by fluorescence emission and total cells by bis-benzimide stained.

Total GSH levels were measured by the method of Tietze [22]. Briefly, the cells from fibroblast cultures were washed with PBS, lysed in 100 µl of 0.4 N perchloric acid (PCA) for 30 min at 4°C, and centrifuged. The supernatants were neutralized with four volumes of 0.1 M NaH2PO4, 5 mM EDTA, pH 7.5. GSH content was measured in a p96 automatic reader by the addition of 5,5-dithio-bis-2-nitrobenzoic acid (DTNB, 0.6 mM), nicotinamide adenine dinucleotide phosphate reduced tetrasodium salt (NADPH, 0.2 mM), and glutathione reductase (1 U). The reaction was monitored at 412 nm for 6 min.

### Measurement of Mitochondrial Reactive Oxygen Species (mROS) and Complex-IV immunoreactivity

Coverslips with adherent fibroblasts were stained with MitoTracker Orange CM-H_2_ TMRos (Gibco, Life-Technologies, Paisley, Scotland, UK) which was prepared in dimethyl sulfoxide and then added to the cell culture medium at a final concentration of 1 µmol/L. After 45 minutes incubation, the cells were analyzed by fluorescence in a Nikon C1 plus ECLIPSE Ti-e microscope. Cells were considered MitoTracker Orange positive if a bright dotted orange fluorescence of the mitochondria was observed and negative if cells exhibited a diffuse orange cytoplasmic staining [23], [24].

Immunocitochemistry of CHIP-mutant fibroblast with anti-complex-IV antibody as a mitochondrial marker was performed (1/200, enzyme cytochrome-c oxidase, Invitrogen) to evaluate the mitochondrial structure and trehalose effect.

### Proteasomal activity measurement

After culture treatments, the cells were washed with PBS, harvested in proteasome lysis buffer and lysed by sonication (VibraCell, level 0.5 for 30 s). The lysates were centrifuged at 12.000 g at 4°C for 30 min. The protein concentration was assayed from the resulting supernatants by the BCA protein assay kit. The 20S proteasomal chymotrypsin-like activity was quantified [25] by monitoring the accumulation of the fluorescent cleavage product 7-amino-4-methylcoumarin (AMC) from the synthetic proteasomal substrate Suc-Leu-Leu-Val-Tyr-aminomethylcoumarin (LLVY-AMC) using the 20S proteasome activity assay kit (Chemicon, Temecula, CA, USA) according to the manufacturer's instructions.

### Autophagy analysis by Immunocytochemistry

We analyzed the percentage of cells immunoreactive to LC3, LAMP-2A, CD63 and HSC70/LAMP-2A colocalization as markers of macro-autophagy and chaperon-mediated autophagy [25], [26]. The fibroblast cultures were fixed with 4% paraformaldehyde, permeabilized with ethanol-acetic acid (19∶1), and incubated at 4°C for 24 h with primary antibodies diluted in PBS containing 10% fetal calf serum. Rabbit polyclonal anti-LC3 antibody (MBL, Nagoya, Japan) 1/200. Mouse anti- HSC70 1/100 and rabbit anti-LAMP-2A 1/100 and were from Abcam (Cambridge, UK). Mouse monoclonal anti-CD63 antibody was from BD Pharmingen (1/100). Fluorescein- and rhodamine-conjugated secondary antibodies were employed to visualize positive cells under fluorescent microscopy. Colocalization images for HSC70 and LAMP-2A were acquired using a Nikon C1 plus ECLIPSE Ti-e confocal microscope with a 60X Plan Apo VC oil objective.

### Western blot analysis

Poly-ubiquinated proteins were measured as previously described [25]. Human skin fibroblasts cultures were treated with epoxomicin (10 nM) or pre-treated with trehalose (50 mM) 15 min before the treatment with epoxomicin for 24 h. For detection of ubiquitinated proteins by Western blot, 15 µg of protein were used to immunoblot assay with a mouse monoclonal antibody to ubiquitin diluted 1/500 from Chemicon (Temecula, CA, USA). Immunoblot of β-actin diluted (1/5000) was performed to demonstrate equal protein loading.

Anti-HDAC6 (Histone Deacetylase 6) was used as CHIP substrate [27]. The chaperone anti-HSP70 and anti-CHIP as well as the autophagy markers: Beclin-1, Atg5Atg12, LC3 and anti-p62, were used as previously described [16], [25]. Fibroblast cultures were homogenized in lysis buffer Tris HCl, with proteases inhibitors. Samples (20–30 µg protein) were added to SDS sample loading buffer, electrophoresed in 10–15% SDS-polyacrylamide gels and then electroblotted to 0.45 µm nitrocellulose membranes, as described by [19].

The antibodies used in the study were the following: the chaperone mouse anti-HSP-70 (1/750), rabbit polyclonal anti-CHIP (1/1000) and goat polyclonal anti-p62 (SQSTM-1) (1/500) were from Santa Cruz (Heidelberg, Germany). Mouse monoclonal anti-β-actin antibody 1/5000 was used as a control of charge after inactivation of nitrocellulose membrane with sodium azide. Anti HDAC6 (1/1000) and anti Atg5-Atg12 (1/500) rabbit polyclonal antibodies were from Abcam (Cambridge, UK). Rabbit polyclonal anti LC3 (1/500) antibody was from MBL Laboratories (Nagoya, Japan). Rabbit polyclonal anti Beclin-1 (1/1000) antibody was from Sigma (Madrid, Spain).

The secondary antibodies (1/1000) followed by ECL detection reagents (Amersham) were used for immunodetection and quantified by computer-assisted video (ImageQuant, GE Healtcare).

### Autophagic flux in control and CHIP-mutant fibroblasts

Cells were treated with 10 µM chloroquine (Sigma), a potent lysosomotropic agent which disrupts lysosomal degradation function, in the presence or absence of trehalose, 24 hours. After treatment, LC3-II accumulation by immunoblotting, LC3, LC3/LAMP2A by immunocitochemistry was performed. To measure the immunocitochemistry data images of 40 cells by cover slip were loaded into Image-Pro software (Media Cybernetics) and the ratios of green alone (FITC, LC3) or with red (rhodamine, LAMP2A) colocalization, yellow, cells were determined by Integrated Optical Density (IOD).

### Statistical analysis

Because this is an individual case based on only one patient the statistical comparisons are not valid between control and patient, control and CHIP-mutant groups were analyzed independently, no inter-group statistical analysis was performed. Data are expressed as the mean ± SEM values of at least two independent experiments. The “n” for the patient group corresponds to pseudo-replicates of different cultures, in the control group it corresponds to the mean of repeated cultures for each one of the three controls (n = 3). Pseudo-replicates occurs when observations are not statistically independent, but treated as if they are, in the CHIP-mutant group we have multiple observations on the same subject [28]. In order to study the effect of different treatments inside the two groups (CHIP-mutant and control fibroblasts) the results were statistically evaluated for significance with Student's t-test. Differences were considered statistically significant when p<0.05. Analysis of data was performed using the GraphPad PRISM 5 software.

## Results

### CHIP expression in mutant fibroblasts and effects of trehalose on levels of chaperones in untreated and epoxomicin-treated CHIP mutant fibroblasts

**Figure 1:**
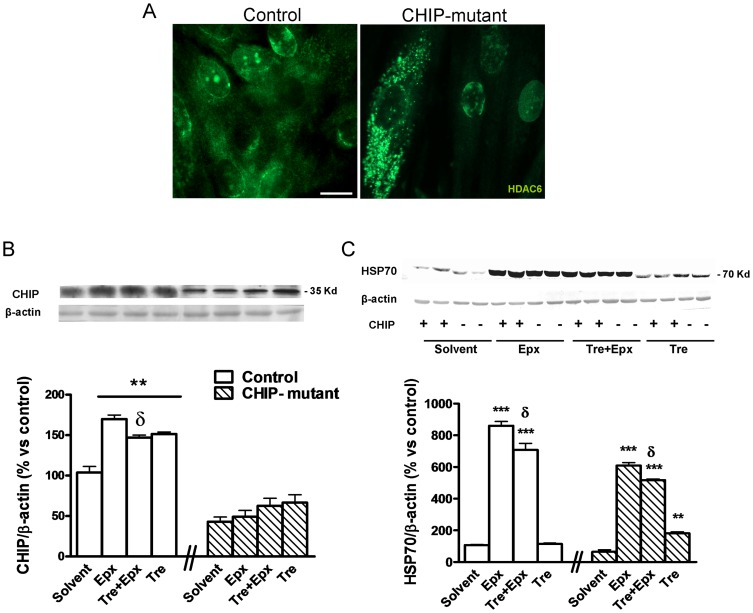
CHIP, HSP70 expression and HDAC6 accumulation in control and CHIP-mutant fibroblasts. Effects of epoxomicin and trehalose on chaperone proteins. After 3 days in vitro, the cells were pre-treated with trehalose (50 mM) for 15 minutes followed by addition of epoxomicin (10 nM) or solvent for another 24 h. **A**) Representative image of control and CHIP-mutant fibroblasts immunostained with antibody to HDAC6 (a substrate of CHIP). **B**) Representative bands and quantification of CHIP and **C**) HSP70 Western blot. β-actin was used as an equal loading of proteins. In 1A, a total of 120 cells in 5 coverslips were analyzed in both groups. Data of the control and CHIP-mutant groups were analyzed independently, no inter-group statistical analysis was performed. Data are expressed as the mean ± SEM values. Values of 1B and 1C correspond to the mean of two experiments with 4 independent cell dishes of 3 different controls (n = 3). In the CHIP-mutant case six independent dishes of one patient (pseudo-replicates, n = 6). Statistical analysis was performed by Student's t-test. *p<0.05, **p<0.01, ***p<0.001 *vs* solvents; δp<0.05 trehalose + epoxomicin *vs* epoxomicin-treated cultures.

There is a clear difference in CHIP levels between control and mutant-CHIP fibroblasts, with a reduced expression in the latter; this may, therefore mimic loss of function. Indeed the mutant fibroblast shows accumulation of HDCA6, a substrate of CHIP [27] (Figure 1A). In the control group an increase in CHIP expression occurs as a stress response to the epoxomicin treatment as well as to trehalose treatment. In this case, trehalose may enhance the basal stress tolerance by regulating the expression of CHIP [29]. Epoxomicin did not change the levels of CHIP in CHIP-mutant fibroblasts. These fibroblasts did not respond to stress conditions. Treatment with trehalose increased the levels of CHIP in 30–50%, with respect to the baseline, in epoxomicin-treated and untreated CHIP-mutant fibroblasts (Figure 1B).

HSP70 is not only a chaperone molecule but also a Heat Shock Protein which responds to stress situations and is regulated by CHIP. Epoxomicin greatly increases the levels of HSP70 in control and CHIP-mutant fibroblasts. Trehalose decreased the levels of HSP70 in both treated epoxomicin groups. Furthermore trehalose treatment significantly increased baseline levels of HSP70 in CHIP-mutant fibroblasts (Figure 1C).

### Cell proliferation in CHIP-mutant fibroblasts and effects of trehalose and epoxomicin on caspase-3 expression

**Figure 2:**
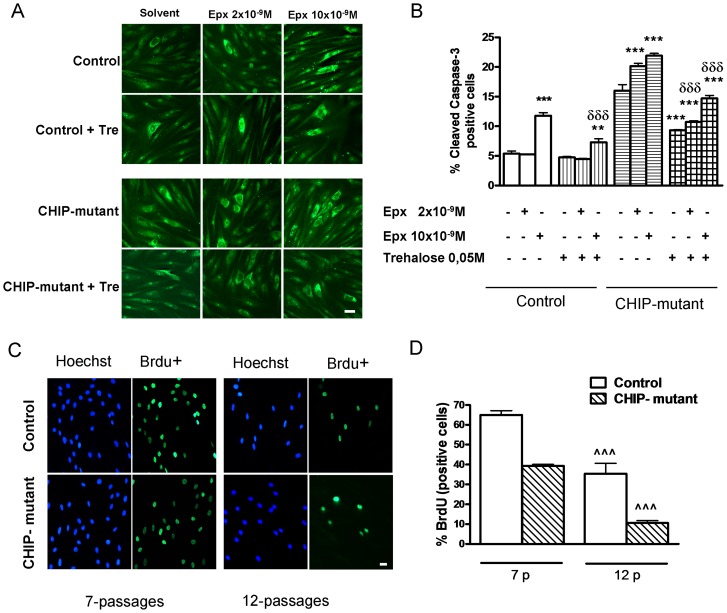
Proliferation and apoptosis of human fibroblasts with compound heterozygous CHIP mutations. Trehalose protects from apoptosis and epoxomicin-induced cell death in CHIP-mutant human fibroblasts. **A**) Photomicrographs of dose-dependent effects of epoxomicin and trehalose pre-treatment 15 min before epoxomicin for 24 h on the expression of cleaved caspase-3 positive cells in control and CHIP-mutant fibroblasts (scale bar  = 30 µm). **B**) Percentage of cleaved caspase-3 positive cells in control and CHIP-mutant fibroblasts treated with 0, 2 and 10 nM epoxomicin for 24 h and trehalose 50 mM pre-treatment after 5 days in vitro. One hundred fifty to two hundred cells per coverslip cells were counted. **C**) Photomicrographs of dividing BrdU^+^ cells and total nuclei stained with bis-benzimide. (Scale bar  = 30 µm). **D**) Percentage of BrdU^+^ cells with respect to the total number of fibroblasts with 7 and 12 passages, respectively. Sixty to eighty cells were counted per coverslip to obtain the percentages of BrdU^+^ cells. Data of the control and CHIP-mutant groups were analyzed independently, no inter-group statistical analysis was performed. Data are expressed as the mean ± SEM. Values are the mean of two experiments of 6 independent cell dishes (pseudo-replicates, n = 6) of the patient group and in control group: the mean of the 3 different controls with 3 dishes of cells each one (n = 3). Statistical analysis was performed by Student's t-test. **p<0.01, ***p<0.001 *vs* solvents; δδδ p<0.001 trehalose + epoxomicin *vs* epoxomicin; ∧∧∧p<0.001 fibroblasts with 12 *vs* 7 passages.

CHIP-mutant fibroblasts show augmented number of cleaved caspase-3 positive cells in a dose dependent pattern by epoxomicin (Figures 2A–2B). Treatment with trehalose 0.05 M greatly reduced by around 40 to 50%, the levels of caspase-3 in both epoxomicin-treated and untreated CHIP-mutant and epoxomicin-treated control fibroblasts.

The central role of the proteasome in senescence and survival of human fibroblasts has been described [7]. As shown in Figures 2C–2D, the proliferating efficiency for both cells gradually decreases as the passage number increase. The number of BrdU^+^ cells at larger (12) number of passages was decreased around 39% and 75% in control and CHIP-mutant fibroblasts respectively.

### Effects of trehalose on free radicals and free radical scavenger GSH in CHIP mutant fibroblasts

**Figure 3:**
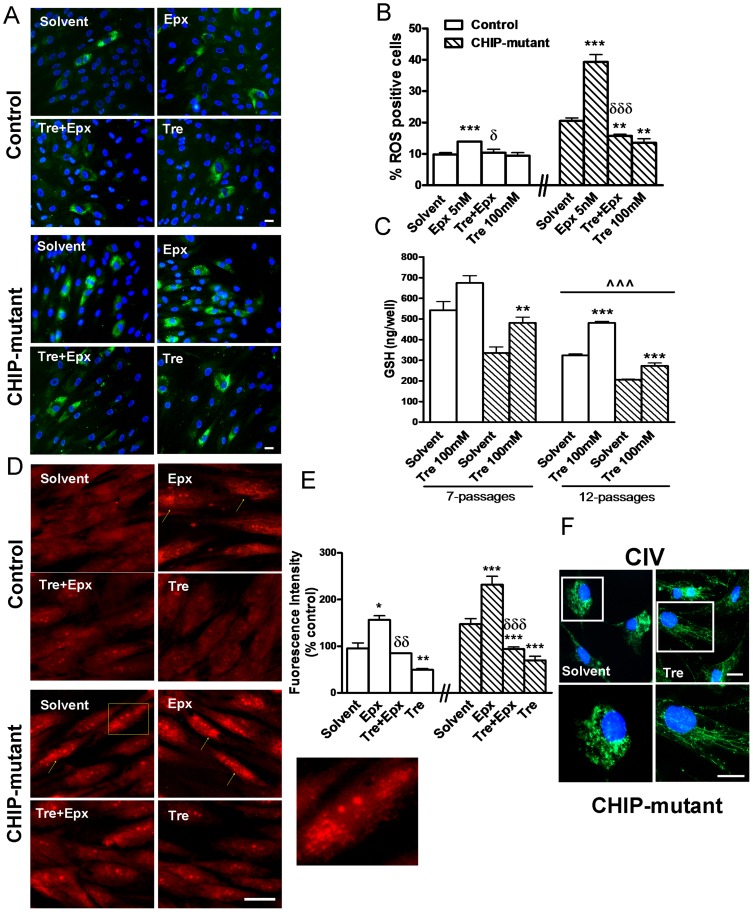
ROS production, Glutathione homeostasis and Mitochondrial membrane potential. **A**) Representative images of DCF staining in control and CHIP-mutant fibroblasts for the indicated treatment. Scale bar  = 10 µm. **B**) Percentage of DCF positive fibroblasts. One hundred fifty to two hundred cells per coverslip cells were counted. **C**) GSH levels in control and CHIP mutant human fibroblasts with 7 and 12 passes. **D**) Representative images of MitoTracker TM Ros and nuclei Hoescht staining in control and CHIP-mutant fibroblasts cultured in the presence of epoxomicin (10 nM) and/or trehalose (50 mM). Yellow arrows indicate small depolarized mitochondria (scale bar  = 30 µm). **E**) Quantification in IOD of fibroblasts with altered mitochondria morphology by fluorescence imaging analysis in 10 random fields of 6 coverslips from control and CHIP-mutant cultures (Scale bar  = 15 µm). **F**) Complex IV staining showed an abundant and granular perinuclear pattern indicating mitochondrial compartmentalization (scale bar  = 10 *µ*m). A cell of this micrograph has been magnified in the pictures below. Data of the control and CHIP-mutant groups were analyzed independently, no inter-group statistical analysis was performed. Data are expressed as the mean ± SEM. Values of 3B are the mean of two independent experiments of 4 independent cell dishes “n = 4” (pseudo-replicates) of the patient group and in control group “n = 2”: the mean of 2 different controls with 4 dishes of cells each one. In 3C and 3E values are the mean ± SEM from two experiments with 6 independent cells dishes (n = 6, pseudo-replicates). Statistical analysis was performed by Student's t-test. *p<0.01, **p<0.01, ***p<0.001 vs solvents; δp<0.05, δδp<0.01, δδδp<0.001 trehalose + epoxomicin vs epoxomicin treated cultures; ∧∧∧p<0.001 fibroblasts with 12 *vs* 7 passages.

To show the oxidative stress provoked by the deficit in chaperone function, the reactive oxygen species (ROS) and GSH intracellular levels were measured in fibroblast cultures. Treatment with epoxomicin increased the levels of free radicals in control and, much more so, in CHIP-mutant fibroblasts. Treatment with trehalose greatly reduced the levels of free radical in control and CHIP mutant fibroblasts, treated or untreated with epoxomicin (Figures 3A and 3B).

Trehalose treatment increased the levels of antioxidant GSH in CHIP mutant fibroblasts at early passages. GSH levels were reduced in both control and CHIP-mutant fibroblasts by successive passages and increased by treatment with trehalose (Figure 3C).

### Effects of trehalose in mitochondrial dysfunction and morphology in CHIP-mutant fibroblasts

MitoTracker Orange has been used in its reduced form, as a marker for mitochondrion ROS production [24]. Staining and imaging analysis revealed discrete diffuse staining of mitochondria in solvent control fibroblast cells, whereas in CHIP-mutant and epoxomicin-treated a punctuated bright staining was observed (Figures 3D–3E).

In order to study the mitochondrial morphology we used an anti-enzyme cytochrome c oxidase antibody, or anti-Complex IV (Figure 3F). This showed mitochondrial network fragmentation, with small, rounded and abundant perinuclear staining in CHIP-mutant fibroblasts.

Trehalose treatment improved mitochondrial morphology and uptake of MitoTracker Orange in these cells, indicating a possible amelioration of the mitochondrial membrane potential (Figures 3D–3F).

### Effects of trehalose on reduction of chymotrypsin-like activity and epoxomicin-induced enhancement of ubiquitin proteins in control and CHIP-mutant fibroblasts

**Figure 4:**
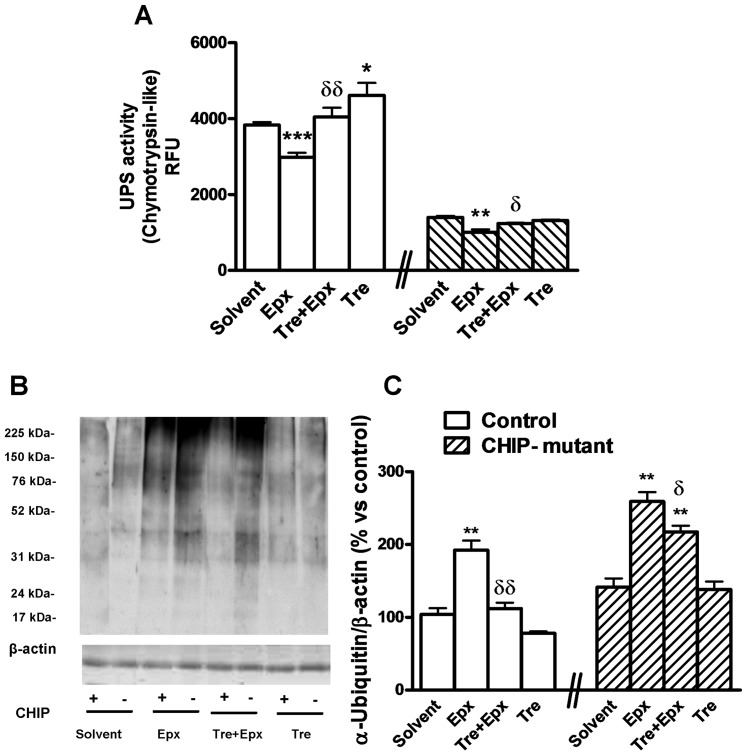
UPS activity in control and CHIP-mutant fibroblasts. Trehalose protects from ubiquitin protein accumulation by epoxomicin in CHIP-mutant and control human fibroblasts. **A**) Differential UPS activity in control and CHIP mutant fibroblasts. **B**) Accumulation of poly-ubiquitinated proteins and **C**) their corresponding densitometry analysis after 24 h of treatment corresponds to the whole smear. Data of the control and CHIP-mutant groups were analyzed independently, no inter-group statistical analysis was performed. Data are expressed as the mean ± SEM. Every value in 4A corresponds to the mean of two experiments: the mean of 4 independent dishes of cells of 3 different controls (n = 3) and for the CHIP-mutant group six independent dishes of one only patient (pseudo-replicates, n = 6). In 4C values are the mean of two independent experiments, with 6 cell dishes (pseudo-replicates, n = 6) of the patient group and, in the control group, the mean of 3 different dishes of cells for each one of the 3 controls (n = 3). Statistical analysis was performed by Student's t-test. *p<0.05, **p<0.01, ***p<0.001 *vs* solvents; δp<0.05, δδp<0.01 trehalose + epoxomicin *vs* epoxomicin treated cultures.

CHIP-mediated stress recovery by sequential ubiquitination of substrates and HSP70 has been published [30]. The epoxomicin treatment produced a decrease in UPS activity, expressed as the chymotrypsin like activity assay, in both control and CHIP-mutant fibroblasts. Co-treatment with trehalose 15 minutes after the epoxomicin addition did not show the reduction of UPS activity seen by the epoxomicin effect, in both control and CHIP mutant fibroblasts (Figure 4A), the levels were similar to solvents nullifying the decline related to epoxomicin.

We used an anti-ubiquitin antibody as an indicator of unfolded protein accumulation. The treatment with epoxomicin exacerbated those poly ubiquitinated proteins in controls and CHIP mutant fibroblasts. Co-treatment with trehalose partially prevented epoxomicin induced increase of ubiquitin (Figures 4B and 4C).

### Effects of epoxomicin and trehalose on autophagy markers in control and CHIP mutant fibroblasts

**Figure 5:**
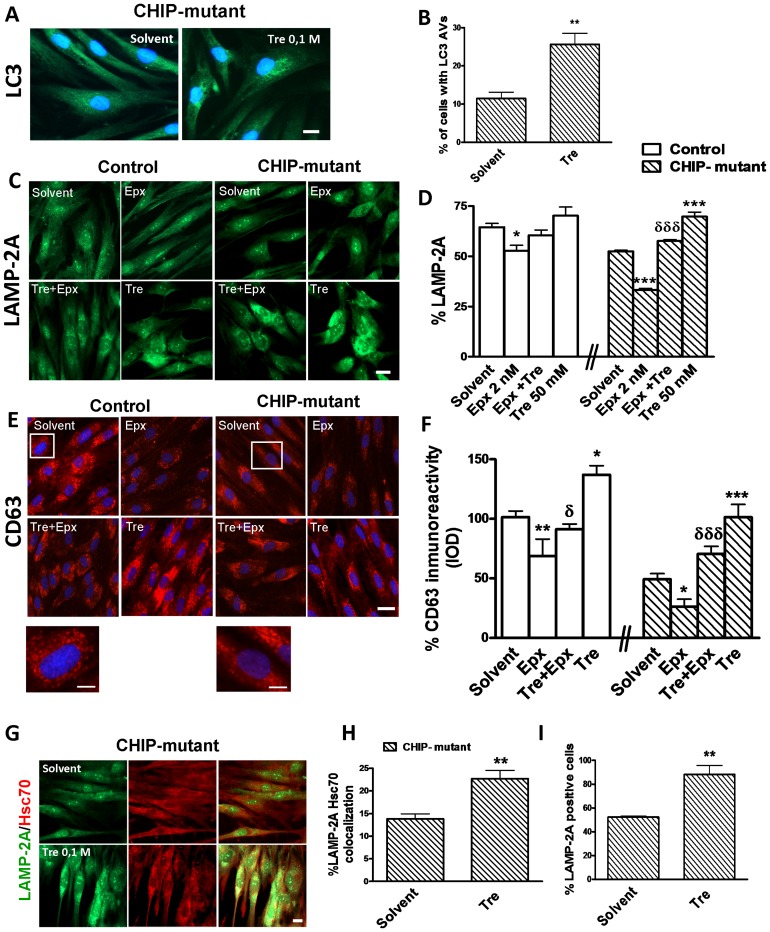
Trehalose increases autophagy activity in CHIP-mutant human fibroblasts. After 5 DIV, fibroblasts were pre-treated with trehalose (50 or 100 mM) for 15 minutes followed by addition of epoxomicin or solvent for another 24 h. **A**) LC3 staining (green) and nuclei (Hoescht, blue) immunofluorescence in CHIP-mutant fibroblast treated with trehalose (100 mM) and their quantification **B**) in percentage of cells with more than 50 autophagic vesicles (Scale bar  = 20 µm). **C**) Representative microphotographs of Lysosomal-associated membrane protein, LAMP-2A, present in lysosomes and endosomes, (Scale bar  = 20 µm) and **D**) quantification in percentage of cells with LAMP-2A positive vesicle distribution in the perinuclear region, around the nucleus. **E**) Images of fibroblasts expressing CD63, as a late endo/lysosomal marker (Scale bar  = 30 µm) and **F**) quantification in percentage of optical intensity for CD63 inmunoreactivity. The insets show boxed regions at higher magnification (Scale bar  = 15 µm). **G**) Immunofluorescence for HSC70 and LAMP-2A in CHIP-mutant cultures treated with trehalose 100 mM for 48 h (Scale bar  = 20 µm) and **H**) percentage of co-localization LAMP-2A/HSC70 positive cells. **I**) Percentage of LAMP-2A positive cells. Data of the control and CHIP-mutant groups were analyzed independently, no inter-group statistical analysis was performed. Data are expressed as the mean ± SEM. Values in 5B, 5D, 5H and 5I are the mean ± S.E.M. of two experiments with six cover slips, from 150 to 250 cells of every group were counted. In 5F, in CD63 immunostaining data are the mean of 4 independent dishes of cells for 2 control individuals (n = 2), in the CHIP-mutant group 6 independent dishes of cells of the only patient (pseudo-replicates, n = 6). Statistical analysis was performed by Student's t-test. *p<0.05, **p<0.01, ***p<0.001 *vs* solvents; δp<0.05, δδδp<0.001 trehalose + epoxomicin *vs* epoxomicin treated cultures.

LAMP-2A and CD63 are two glycosylated membrane proteins localized in vesicles of the late endosome and lysosome, therefore these two are considered as markers of mature autophagy in the limiting membrane of the cytoplasmic vacuoles in cultured fibroblasts. Epoxomicin treatment reduced the number of LAMP-2A and CD63 positive cells in both control and CHIP mutant fibroblasts. Co-treatment with trehalose restored LAMP-2A and CD63 immunoreactivity, confirming the strong effect of trehalose in autophagy activation (Figure 5C–5F).

In order to study the implication of the CMA we analyzed the percentage of double LAMP-2A/HSC70 positive fibroblast in the solvent and trehalose-treated CHIP-mutant fibroblasts. We observed an increase in the percentage of co-localization of both antibodies due to trehalose, implicating the CMA in the trehalose effect (Figures 5G and 5H).

**Figure 6:**
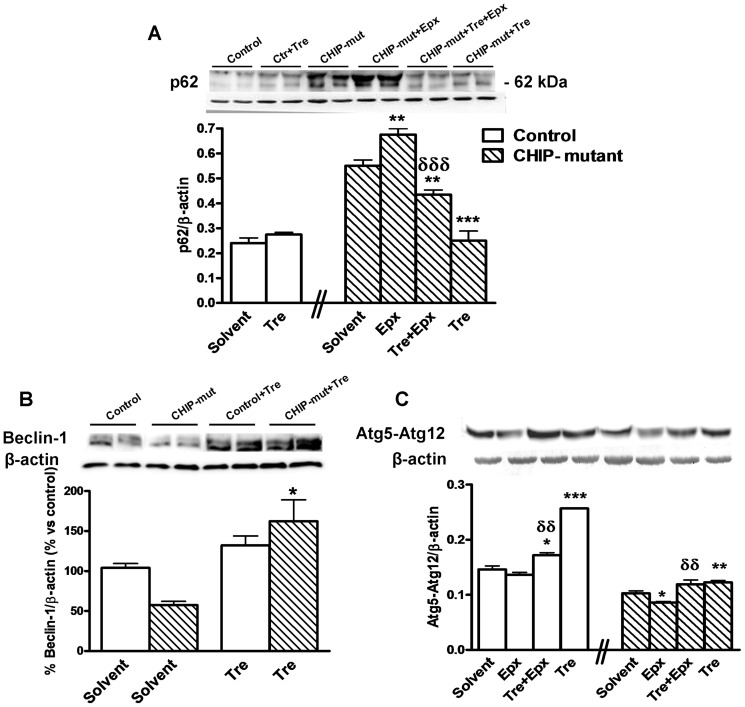
Expression of p62, Atg5–Atg12 and Beclin-1 in controls and CHIP-mutant fibroblasts. Trehalose treatment increases the autophagic activity. Fibroblasts were exposed to 50 mM trehalose for 24 hours and the autophagy-related proteins were analyzed by Western blot. **A**) Western blot showing the autophagic degradation of p62, a major selective substrate for autophagy. **B**) Beclin-1 and **C**) Atg5-12, essential factors for autophagosome formation were increased by trehalose. Data of the control and CHIP-mutant groups were analyzed independently, no inter-group statistical analysis was performed. Data are expressed as the mean ± SEM. Values are the mean of two experiments of independent dishes of one patient (pseudo-replicates, n = 4–6) and for control group 3 different controls with the mean of different dishes of cells (n = 3). Statistical analysis was performed by Student's t-test. *p<0.05, **p<0.01, ***p<0.001 *vs* solvents; δδp<0.01, δδδp<0.001 trehalose + epoxomicin *vs* epoxomicin treated cultures.

The SQSTM1/p62 protein (hereafter referred to as p62), an endogenous autophagic substrate, is widely used to monitor the activation of autophagy. An accumulation of p62 was observed in CHIP-mutant fibroblasts and even more by epoxomicin treatment. This accumulation was also greatly reduced with trehalose treatment in epoxomicin-treated and untreated CHIP mutant fibroblasts (Figure 6A). This result suggests that the treatment with trehalose increases the elimination of these protein aggregates. In support of that, we found that the levels of Beclin-1 and Atg5-Atg12 conjugate, essentials to autophagosome formation (Figure 6B and 6C) [31] were also decreased in epoxomicin treated CHIP-mutant fibroblasts as well as LC3 and LAMP-2A (Figures 5A–5D). These direct markers of mature autophagy were increased by trehalose.

### Changes in autophagy turnover in control and CHIP-mutant fibroblasts

**Figure 7:**
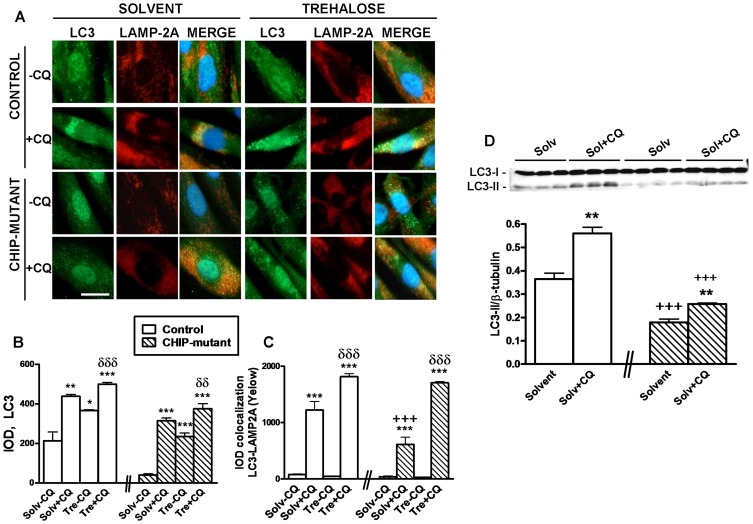
Trehalose induces autophagy and activates autophagic flux in CHIP-mutant fibroblasts. The cells were treated for 24 h with o without trehalose 50 mM in the presence or absence of 10 µM chloroquine (CQ), a lysosomotropic agent widely used to block lysosomal degradation. **A**) Fluorescence microscopy images showing autophagosome marker LC-3 (green), lysosomal marker Lamp-2A (red) and LC3-LAMP-2A colocalization (yellow). (Scale bar  = 20 µm). **B**) Integrated optical density (IOD) quantification of LC3 expression. **C**) The lysosomal degradation was measured by IOD quantification of colocalization LC-3-LAMP-2A (yellow). **D**) Western blot showing LC3-II accumulation. Data of the control and CHIP-mutant groups were analyzed independently, no inter-group statistical analysis was performed. Data are expressed as the mean ± SEM. In 7B and 7C values are the mean ± S.E.M. of six independent coverslips (each value represents the mean of 20 field for coverslip). Three individuals with the mean of 2 independent coverslips (n = 3) in the control group and in the CHIP-mutant group 6 independent coverslips of the only patient (pseudo-replicates, n = 6). In 7D values represents the mean of 2 independent dishes of cells of 2 controls (n = 2) and for the CHIP-mutant group one patient with four independent dishes (pseudo-replicates, n = 4). Statistical analysis was performed by Student's t-test. *p<0.05, **p<0.01, ***p<0.001 *vs* solvents without chloroquine; δδp<0.01, δδδp<0.001 trehalose -CQ *vs* trehalose + CQ treated cultures.

To measure autophagic flux we monitored the LC3 turnover through observation of LC3 degradation in autolysosomes and its accumulation by Western blot [32]. Fibroblast cultures were treated with or without trehalose in absence or presence of CQ (10 µM). CQ suppresses the capacity of lysosomal degradation by increasing lysosomal pH and thus suppressing autophagy. CQ alone increased LC3 expression in both cells types (Figure 7A–7D). Additionally when we studied the LC3-LAMP-2A colocalization, we found that CQ-treated control and CHIP-mutant fibroblasts had decreased the IOD intensity for yellow puncta respect to trehalose + CQ treatment (Figure 7A–7C), suggesting that trehalose causes an increase in the autophagic flux. Interestingly LC3 puncta was increased by trehalose or CQ treatment. The study of lysosomal degradation of LC3 by LAMP2 and LC3 colocalization confirms these results (Figure 7C), so trehalose activates autophagy in both, control and CHIP-mutant fibroblasts.

## Discussion

In this work we describe cellular and functional characteristics from a patient with a new CHIP-related ataxia (described by Bettencourt et al. currently submitted to PLoS ONE) and how they respond to trehalose treatment in vitro, regulating protein homeostasis. Furthermore we studied the different sensitivity after blocking the proteasome activity with epoxomicin, and how trehalose could protect this effect and even revert some of the baseline pathologies. This patient fibroblasts could present alterations according to pathological changes related to CHIP deficiency as: proteasome deficits, augmented apoptosis, protein accumulation [2], [6], [33], [34] and ROS reactivity [35]. Trehalose treatment *in vitro* reverted most of the epoxomicin abnormalities observed in both control and mutant fibroblast. Furthermore, trehalose increased the autophagy markers, HSP70 co-chaperone expression and recovered ROS solvent levels in CHIP-mutant fibroblasts.

The dimerization of CHIP is crucial for its function, and is dependent on strongly conserved residues that promote distinct dimer contacts. The second mutation found in this family (c.633G > A, p.Met211Ile) corresponds precisely to one of those residues of the second dimer interface [5] and, therefore, may affect the formation of CHIP dimers. Abnormal CHIP function may lead to neuronal dysfunction and loss of Purkinje cells as it has been observed in one case from this family [4] and as it happens in other hereditary ataxias such as SCA3 [36]. CHIP may also play an important role in other neurodegenerative disorders since it is of paramount importance in the ubiquitination of proteins like tau, alpha-synuclein and others [37].

CHIP is a 3-ubiquitin ligase that marks proteins to ubiquitin proteasomal system degradation. In addition there is evidence for CHIP and HSP70 acting together as peptide clearance promoters, where the CHIP acts as a co-chaperone protein [38]. Here, we found that the treatment of the fibroblasts with trehalose increased the levels of CHIP. We do not believe that the improvement of CHIP mutant fibroblasts is related to this elevation of CHIP levels since, as discussed above, the second mutation will probably, in spite of the lack of functional studies, make CHIP non functional.

HSP70 is the major stress-inducible chaperone with broad neuroprotective properties under conditions of oxidative stress, mitochondrial dysfunction and chronic insults. HSP70 is an inducible protein in stress situations and is as well considered as a cell damage biomarker, but it is also a chaperone that helps in the correct protein folding and transport [39], [40]. Following a mild stress the expression of HSP mRNA dramatically increases. During a prolonged stress activity could return rapidly to near-constitutive levels. Recent studies show that there is regulation of HSPs through trehalose in yeast [41], a high trehalose level prevents the HSP70 decrease during a sustained heat shock response and other works link the age and HSP70 expression. These works showed a down regulation of HSP70 and other reactive proteins to stress [42] due to a more sensitive state in the elderly, which is probably caused by accumulation of abnormal proteins [43].

In our results, we saw a clear increase of HSP70 with epoxomicin treatment in both groups, probably due to the acute insult that supposes the mitochondrial inhibitor, but interestingly trehalose reduces the overexpression of epoxomicin treatment and increases the basal expression of HSP70 of CHIP-mutant fibroblasts. In our work trehalose could be acting as a HSP70 stabilizer, could normalize HSP70 expression and may improve clinical deficits related with CHIP dysfunction. Perhaps this regulation is made by diminishing the damage by other methods and reducing the cellular stress [29], [44], [45].

Probably in CHIP-mutant fibroblasts, the failure in ubiquitination of CHIP-substrate proteins or a non-functional HSP70-CHIP complex in its chaperone activity could show proteasome activity deficits. This double clearance protein malfunction increased the accumulation of misfolded proteins and enhanced the aged-senescence phenotype [8], [46]. Taken all together, our data supports the role of protein quality control mediated by CHIP on cellular aging. Trehalose showed a recovery of the epoxomicin effects in proteasome activity, probably mediated by the clearance of polyubiquitin proteins overload through the autophagy system [7], [8], [47]. The data presented here demonstrated that primary fibroblasts from this patient with *STUB1* mutations had reduced expression of the CHIP protein, this dysfunction produce a series of CHIP-related pathologies as high senescence and low proliferation in this fibroblasts [33]. It is known that CHIP is a negative regulator for TGF-β and that silencing CHIP expression led to increased TGF-β signalling sensitivity mediating in cell cycle arrest [48]. Furthermore, when CHIP-mutant fibroblasts were exposed to epoxomicin, there was a marked increase in the percentage of apoptotic cells. These findings suggest that apoptosis can be significantly attenuated by therapeutic blockade of stress processes with trehalose. The pro-survival role of autophagy is confirmed by mTOR-independent pro-autophagic trehalose, which protected from CHIP-mutant and epoxomicin induced apoptosis.

A number of studies have demonstrated that the redox system is associated to cellular senescence. Proteasome degradation of oxydized proteins, act as a cellular defence system [49]. We saw a direct correlation between CHIP-mutant and oxydative stress: ROS were augmented and GSH levels were decreased in CHIP-mutant, more pronounced after inhibition of proteasome with epoxomicin. Both additive effects are reverted by trehalose. The GSH levels are increased by trehalose even in the aged fibroblasts. Increased ROS production and oxidative stress is a common consequence of mitochondrial dysfunction. Our results with the MitoTracker Orange and Complex-IV immunostaining showed an increase in ROS mitochondrial production and altered mitochondrial structure, with a large percentage of mitochondrial population disorganized in CHIP-mutant fibroblasts. This reaction was augmented with epoxomicin-treatment in both, control and mutant fibroblasts. There are studies showing trehalose preserving the mitochondrial membrane from various insults [50], even linking it with the mitochondrial chain regulation itself [51], and that autophagy induction with rapamycin leads to protection via the mitochondrial cell death pathway [52]. As far as we know, the beneficial effects of trehalose on CHIP-mutant fibroblasts seem to be mostly mediated by the enhancement of autophagy. Furthermore due to its anti-aggregation properties, trehalose is also an autophagy enhancer as previously described [12], [53].

A very interesting point of our work is the improvement in mitochondrial morphology with trehalose, showing a tubular morphology of the mitochondrion and a reduction in the mitochondrion generated ROS. This recovery may be explained by the elimination of dysfunctional mitochondria (mitophagy) and mitochondrial misfolded protein overload (CMA and/or Macroautophagy), together with the protection of the mitochondrial membrane integrity by trehalose.

The chaperone system is intimately associated with the ubiquitin-proteasome system and the autophagy-lysosomal pathway. Both are responsible for the elimination of misfolded protein, protein quality control and regulation of proteostatic stress response in aging. We saw a decrease in LAMP-2A and CD63 in CHIP-mutant fibroblasts compared to trehalose-treated group and as a consequence, we speculate that mitochondrial dysfunction due to CIV respiratory chain complex blockade could decrease the autophagic capacity of the affected fibroblasts, and result in premature cellular ageing [54], [55]. UPS inhibition may induce neurodegeneration and it could be reverted by increasing autophagy [56]. Changes in p62 were observed with epoxomicin, showing an accumulation of this autophagy substrate, but again a remarkable decrease after trehalose treatment in mutant-CHIP fibroblast was observed. In addition we saw four direct markers of the autophagy process (Beclin-1, Atg5-Atg12 complex, LC3 II and LAMP-2A) augmented significantly with trehalose. Also we found higher co-localization of HSC70 and LAMP-2A, two proteins related to CMA activation and CD63 as late endo/lysosomal marker. Induction of autophagy by trehalose improved UPS malfunction by epoxomicin as well as viability problems, autophagy failure and ROS generation effects because of CHIP mutations.

The process of autophagy is complex and it has at least three inter-related mechanisms: proteasome mediated (PMA), chaperone mediated (CMA) and macro-autophagy. Macro-autophagy is short lasting and takes place in the first hours, while CMA starts later and is more prolonged [57], [58], and we found markers of these two types in our results increased by trehalose treatment. Nevertheless the prolonged up-regulation of autophagy pathway has to be treated with caution because enhanced mitochondrial turnover could be harmful [59]. However it could be possible to induce the autophagy in proteinopathies, reducing the mitochondrial load with substantial beneficial effects without affecting the respiration [12], [47], becoming very effective in reverting oxidative stress conditions [60].

We underline that genetic, biochemical and functional fibroblasts studies are helpful in clarifying CHIP-mutations effects detected on this ataxia patient. Beyond the obvious problem of the only one patient analysis, our study endorses the hypothesis that trehalose, due to its effects on protein quality control, lack of secondary effects as a disaccharide and the oral bioavailability might be effective in treating the pathologies seen in this new case of ataxia. Accordingly there is a rational base for the implementation of well-designed clinical trials in groups of STUB1 gene patients, with autosomal dominant, autosomal recessive or even sporadic resembling ataxias, as more and more families are discovered with mutations impairing CHIP function.
